# Microarray analysis of long non-coding RNA expression profiles uncovers a *Toxoplasma*-induced negative regulation of host immune signaling

**DOI:** 10.1186/s13071-018-2697-8

**Published:** 2018-03-12

**Authors:** Wenquan Liu, Liyang Huang, Qimei Wei, Yu Zhang, Shengnan Zhang, Wenting Zhang, Liya Cai, Shaohui Liang

**Affiliations:** Department of Parasitology, Wenzhou Medical University, Wenzhou, Zhejiang Province China

**Keywords:** *Toxoplasma gondii*, Long non-coding RNA, Microarray, UNC93B1, Immune signaling

## Abstract

**Background:**

*Toxoplasma gondii* is an obligate intracellular protozoan parasite that can infect mammalian cells and thereby regulate host gene expression. The long non-coding RNAs (lncRNAs) have been demonstrated to be an important class of RNA molecules that regulate many biological processes, including host-pathogen interactions. However, the role of host lncRNAs in the response to *T. gondii* infection remains largely unknown.

**Methods:**

We applied a microarray approach to determine the differential expression profiles of both lncRNAs and mRNAs in the human foreskin fibroblast (HFF) cells after *T. gondii* infection. The Gene Ontology and Kyoto Encyclopedia of Genes and Genomes (KEGG) pathway analyses were performed to reveal the potential functions of *T. gondii*-induced genes. Based on the co-expression networks of lncRNAs and immune-related genes, the role of NONSHAT022487 on the regulation of UNC93B1 related immune signaling was investigated by the knockdown and over-expression of lncRNA in human macrophage derived from the PMA-induced promonocytic cell line THP-1.

**Results:**

Our data showed that 996 lncRNAs and 109 mRNAs in HFF cells were significantly and differentially expressed following *T. gondii* infection (fold change ≥ 5, *P* < 0.05). The results from the GO and KEGG pathway analyses indicated that the mRNAs with differential expression were mainly involved in the host immune response. Remarkably, we identified a novel lncRNA, NONSHAT022487, which suppresses the expression of the immune-related molecule UNC93B1. After *T. gondii* infection, NONSHAT022487 impaired the secretion of the cytokines IL-12, TNF-α, IL-1β and IFN-γ by downregulating UNC93B1 expression in human macrophage cells.

**Conclusions:**

Our study identified infection-induced lncRNA expression as a novel mechanism by which the *Toxoplasma* parasite regulates host immune signaling, which advances our understanding of the interaction of *T. gondii* parasites and host cells.

**Electronic supplementary material:**

The online version of this article (10.1186/s13071-018-2697-8) contains supplementary material, which is available to authorized users.

## Background

*Toxoplasma gondii* is an obligate intracellular protozoan parasite that can infect most species of warm-blooded animals around the world [1, 2]. *Toxoplasma gondii* infection in immunocompetent adults is often subclinical and persists in the life of the host [3]. It can also cause several serious diseases, such as neonatal mortality and fetal infection, which occur in the congenitally infected infants and immunocompromised patients [1, 3, 4]. Manipulating the host environment is a critical step for *T. gondii* to establish its successful invasion and survival in host cells [5–7]. It injects parasite-derived effector molecules into the host cell to interfere with their defenses during the invasion [8]. Immediately following the invasion, *T. gondii* establishes a specialized parasitophorous vacuole (PV) within the host cell cytoplasm [9]. The PV protects the parasites against lysosomal destruction and provides a residence in which the parasites can replicate within the host cells [10]. Meanwhile, the intracellular parasites can change the host biological process to maintain its persistence, such as by inhibiting apoptosis [11, 12], inducing autophagy [13], controlling the cell cycle [14] and regulating immune signaling [15].

Long non-coding RNAs (lncRNAs), which have a length of longer than 200 nucleotides and lack a protein-coding capacity, represent a significant proportion of the human transcriptome [16]. Thousands of mammalian lncRNAs have identified their regulatory function in various biological processes, including cell development [17], chromatin modification [16] and immune regulation [18]. LncRNAs have gained great interest for their wide variety of regulatory roles [19]. They can interact with RNA, DNA, protein or microRNAs to regulate transcription, splicing, nucleic acid degradation and translation [16, 20]. The dysregulation of lncRNA expression can lead to cell functional deficiencies that contributes to a variety of diseases, such as developmental defects [21], tumorigenesis [22] and autoimmune diseases [23]. Recent studies have shown that viral or bacterial infection can change the expression profiles of lncRNAs in the host, which indicated that lncRNAs are involved in the regulation of host-pathogen interactions, and even determine the outcome of infection [24–27]. Several lines of emerging evidence have demonstrated that small non-coding RNAs (microRNAs) are involved in the host-*T. gondii* interaction [28–31]. However, the role of lncRNAs in the response to the *Toxoplasma* parasite is still unclear.

In this study, we explored the differential expression profiles of both lncRNAs and mRNAs in human foreskin fibroblast (HFF) cells after *T. gondii* infection. Our data showed that the differentially expressed mRNAs were correlated with host immune signaling. We also identified an lncRNA, NONSHAT022487, which suppresses UNC93B1-related immune signaling in *T. gondii*-infected human macrophage cells. Our study reveals a role of lncRNAs induced by *T. gondii* infection in regulating host immune responses, which advances our understanding of the interaction of *T. gondii* parasites and host cells.

## Methods

### Cell culture

The human foreskin fibroblast cell line (HFF, CRL-2522) and THP-1(TIB-202) cells were obtained from ATCC, and the human embryonic kidney 293 cell line was kindly provided by Jianming Wu (Wenzhou Medical University, Wenzhou, China). HFF and 293 cells were cultured in Dulbecco’s Modified Eagle Medium (DMEM) supplemented with 10% heat-inactivated fetal bovine serum (FBS, Gibco, LA, USA), 2 mM L-glutamine, penicillin (100 U/ml) and streptomycin (100 μg/ml). THP-1 is a human promonocytic cell line that was used as the in vitro model for studying *T. gondii-*induced immune modulation [32]. THP-1 cells were cultured in RPMI 1640 medium (Gibco, LA, USA) supplemented with 10% FBS, 2 mM L-glutamine, penicillin (100 U/ml) and streptomycin (100 μg/ml). Prior to parasite infection, THP-1 cells were plated in 6-well flat-bottom plates (Falcon, NY, USA) and primed with 0.5 μM PMA (Sigma-Aldrich, Boston, MA, USA) for 3 h. The primed cells were then washed with 1640 medium and allowed to adhere overnight to generate macrophages. The THP-1-derived macrophages were used for subsequent experiments. HFF, 293 and THP-1 cells were maintained at 37 °C and 5% CO_2_.

### Parasite infection

The ME49 strain of *Toxoplasma gondii* was multiplied in HFF cells as previously described [33]. Briefly, the *T. gondii* ME49 strain was multiplied in HFF cells and cultured in DMEM containing 10% FBS and 2 mM L-glutamine, penicillin (100 U/ml) and streptomycin (100 μg/ml). The tachyzoites were isolated from the infected HFF monolayers using syringe lysis and washed with DMEM twice. The tachyzoite cultures were further purified using a 5 μm pore size polycarbonate membrane (Millipore, Bedford, MA, USA) to remove the host cell debris, and resuspended in DMEM with 10% FBS for use in the following infections.

HFF cells were infected with purified tachyzoites with a multiplicity of infection (MOI) of 0.2. Six h after parasite inoculation, the cultures were washed twice with PBS to remove any non-adherent *T. gondii* and cultured at 5% CO_2_ and 37 °C for 48 h. Both uninfected and inactivated *T. gondii*-treated HFF cells were used as controls. *Toxoplasma gondii* parasites were inactivated in a 60 °C water bath for 20 min. The death of the parasites was then tested by in vitro passage in HFF cells. Cell samples were harvested and stored at -80 °C for subsequent analysis.

### RNA preparation

Total RNA was extracted from different cell sample using TRIzol Reagent (Invitrogen, CA, USA), according to the manufacturer’s protocol. The RNA was cleaned up with an RNasey Mini Kit (Qiagen, Boston, MA, USA) and then treated with DNase I (Invitrogen) to remove the genomic DNA. The RNA quality and quantity were assessed by denaturing agarose gel electrophoresis and a Du530 spectrophotometer (Beckman, Heidelberg, Germany).

### Microarray analysis

The samples for the microarray were prepared according to the protocol by the manufacturer (Agilent Technology, Santa Clara, CA, USA). One hundred ng of total RNA from each sample was amplified and transcribed into fluorescent cDNAs using the random primer method. The labeled cDNAs were purified by an RNeasy Extraction Kit (Qiagen). Human lncRNA array V4.0 (Arraystar, Rockville, MD, USA), which contains 40,173 human lncRNAs and 20,730 mRNAs, was employed in this study. The Agilent Array Software v.10.7 was used for the microarray analysis. The microarray hybridization was performed by Lian chuan Bio-tech (Shanghai, China). Each sample was performed with three biological replicates. Both differentially expressed lncRNAs and mRNAs with statistical significance between the two groups were identified through *P*-value, false discovery rate (FDR) and fold-change filtering. The lncRNAs and mRNAs with Fold change ≥ 2 (*P* < 0.05) were considered statistically significant in microarry analysis. The microarray data have been deposited in the NCBI Gene Expression Omnibus (GEO), and the GEO accession number is GSE92603. Hierarchical clustering and combined analyses were performed by Lian chuan Bio-tech (Shanghai, China) using Agilent GeneSpring GX software (version 11.5.1).

### Real-time quantitative PCR analysis

Following RNA extraction, the first-strand cDNA was synthesized using the PrimeScript RT Reagent Kit with M-MLV reverse transcriptase (Invitrogen) according to the protocol. Real-time quantitative PCR (RT-qPCR) was performed using Master Mix (Promega, San Luis Obispo, CA, USA) on an ABI7300 Fluorescent Quantitative PCR (Applied Biosystems, Foster City, CA, USA). The RT- qPCR cycles were: 95 °C for 30 s, followed by 40 cycles at 95 °C for 5 s and 60 °C for 5 s. GAPDH was included as an internal control. The relative levels of gene expression were calculated using the 2^-ΔΔ^Ct method. Each sample was repeated in three independent experiments. The primers for the lncRNAs and mRNAs (Additional file 1: Table S1) were designed and synthesized in BGI (Shanghai, China).

### Bioinformatics analysis

Gene ontology (GO) analysis was performed to identify the potential biological functions based on the differentially expressed mRNAs (www.geneontology.org). It covers three classifications, including biological processes, cellular components and molecular functions. Fisher’s exact test was applied to classify the GO categories. Enrichment scores were calculated based on log_10_ (*P*-value). The lower the *P*-value, the more significant the GO term (*P* < 0.05 is required). The Kyoto Encyclopedia of Genes and Genomes (KEGG) pathway analysis (http://www.kegg.jp) was also adopted to map the differentially expressed genes. The enrichment and statistical calculations were similar to the GO analysis. *P* < 0.05 was considered statistically significant. The co-expression network of the lncRNA and mRNA was constructed using Cytoscape software.

### shRNA plasmid construction and knockdown

ShRNA knockdown was performed using adenovirus transduction as described previously [34]. The small hairpin RNA (shRNA) for lncRNA NONHSAT022487 (Transcript ID: NONHSAT022487.2, http://www.noncode.org) was synthesized (GenePharma, Shanghai, China). The shRNA targeting sequence for NONHSAT022487 is 5′-CCA ATA CCG CAG GGT CTC CGA-3′. The shRNA sequence for GFP (5′-CCC GCA AGC TGA CCC TGA AGT TCT TCA AGA GAG AAC TTC AGG GTC AGC TTG CTT TTT GGA AA-3′) was included as a control. The shRNA sequences were cloned into a shuttle vector pHBAd-U6-GFP (Hanheng, Shanghai, China) and were co-transfected with the backbone vector pBHGlox(delta)E1,3Cre into 293 cells to package the recombinant adenovirus using Lipofectamine 2000 (Life Technologies, Carlsbad, CA, USA) according to the manufacturer’s protocol. After 10 days of culture, the cells were collected, frozen, thawed twice at temperatures between -70 °C and 37 °C, and centrifuged at 2000× *g* for 5 min at 4 °C. The first-generation recombinant adenovirus in the supernatant was collected and used to infect 293 cells for two rounds of propagation. Then, high-titer recombinant adenovirus was harvested and stored at -80 °C until use.

For knockdown studies, HFF and THP-1 monocytes were seeded at 1 × 10^6^ cells per well in a 6-well plate, individually. The next day, the cells were infected with recombinant adenovirus expressing shNONHSAT022487 (sh-NONHSAT022487), and cultured for 48 h. Infection efficiency was monitored by observing the number of GFP-positive cells under a fluorescence microscope. The cell samples were harvested for RT-qPCR and Western blotting. The adenovirus-expressing shRNA for GFP was taken as the control (sh-control).

### Overexpression of NONHSAT022487

For overexpression of NONHSAT022487, the sequence of NONHSAT022487 was amplified using the following primers: forward (5′-AAG TCT AGA GAG AGG GTG CAC GTG ACG CTG G-3′) and reverse (5′-CGG GGT ACC TTA AAG AAG GAA GAG GTT TAT TCA GC-3′). Then, the NONHSAT022487 gene was inserted into the multiple cloning site of pcDNA3.1(-) using *Xba*I and *Kpn*I restriction endonucleases. The recombinant vector pcDNA3.1-NONHSAT022487 (pc-NONHSAT022487) was confirmed by sequencing in BGI (Shanghai, China). HFF and THP-1 cells were transfected with the pc-NONHSAT022487 construct using the TransIT®-LT1 Transfection Reagent (Mirus Bio., Madison, WI, USA) following the instructions by the manufacturer. The pcDNA3.1 empty plasmid was included as a control (pc-control). After 48 h of transfection, the cells were harvested for qPCR and Western blotting.

### Western blotting

Cells were lysed in RIPA buffer (Beyotime, Shanghai, P.R. China) supplemented with a protease inhibitor cocktail (Roche, Foster City, CA, USA). The concentration of proteins was detected using an enhanced BCA Protein Assay kit (Beyotime). Equal amounts of protein samples were submitted to SDS-PAGE and Western blotting. Briefly, the proteins were transferred onto PVDF membranes (Bio-Rad,Hercules, CA, USA) and blocked with 5% non-fat dry milk in TBST buffer (25 mM Tris-HCl, 125 mM NaCl, 0.1% Tween 20) for 2 h at 37 °C. Then, the rabbit anti-humanUNC93B1 primary antibody (Abcam, Cambridge, MA, USA) was incubated at 4 °C overnight. After washing 3 times with TBST buffer, the membranes were incubated with Peroxidase-conjugated anti-rabbit IgG secondary antibody (Abcam) for 1 h. GAPDH was included as an internal control. The Western blots were visualized, and the densitometric measurements of the band intensity was performed using Quantity One software.

### Cytokine ELISA

THP-1 cells were primed in the macrophage with 0.5 μM PMA, and then were infected by adenovirus shNONHSAT022487 or transfected with pcNONHSAT022487 construct. After 6 h of infection or transfection, the culture medium was replaced with fresh medium, and the supernatant was collected after 48 h by centrifugation at 3000× *rpm* for 20 min at 4 °C. For *T. gondii* infection, the PMA-induced THP-1 cells were incubated with *T. gondii* at an MOI of 1 after infection with shNONHSAT022487 or transfection with pcNONHSAT022487 plasmid for 24 h. The shRNA-GFP and pCDNA3.1 empty plasmids were taken as controls, respectively. The culture medium was replaced with fresh medium after *T. gondii* infection for 6 h. After culturing for 48 h, the supernatant was collected by centrifugation. Three biological replicates were performed for each sample. The levels of IL-12, TNF-α, IL-1β and IFN-γ were measured, respectively, using commercially available ELISA kits (R&D Systems, Minneapolis, MN, USA) according to the instructions by the manufacturer. Cytokine concentrations in the samples were calculated using standard curves generated from recombinant cytokines, and the results were expressed in picograms per milliliter.

### Statistical analysis

Statistical analyses were performed using SPSS 17.0 software package. All results were expressed as the mean with standard deviation (SD) from three independent biological replicates for each treatment. Student’s t-test was applied to compare the difference between the two groups. Moreover, the FDR was calculated to correct the *P*-value. *P* < 0.05 was considered statistically significant.

## Results

### Differential expression of lncRNAs after *T. gondii* infection

**Fig. 1:**
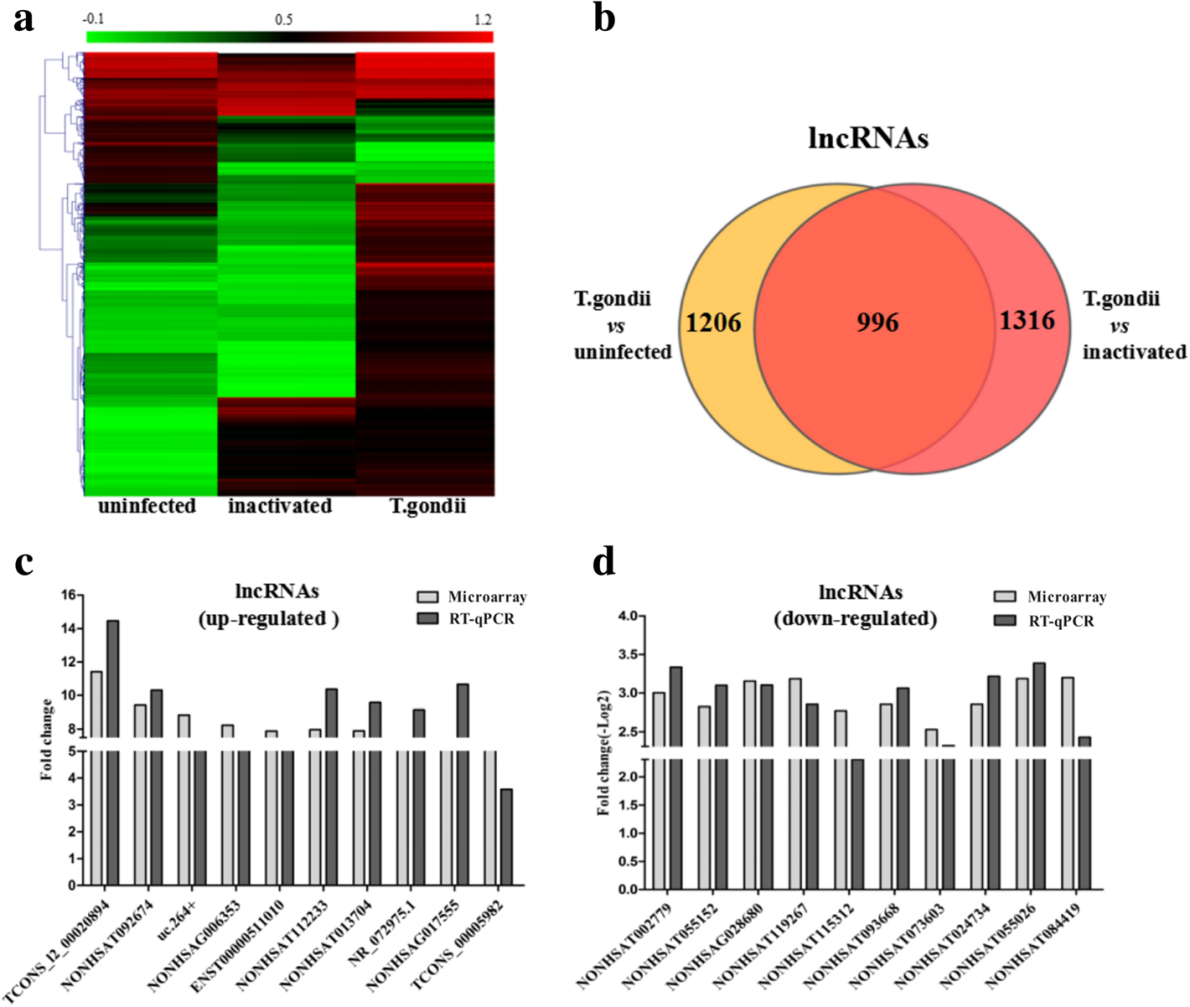
Differential expression of lncRNAs after *T. gondii* infection. **a** The hierarchical clustering of significantly differentially expressed lncRNAs (fold change ≥ 5, *P* < 0.05) between the uninfected HFF cells (uninfected), the inactived *T. gondii*-infected HFF cells (inactivated) and the *T. gondii*-infected HFF cells (*T. gondii*). In the heat map, red indicates the high relative expression, and green indicates the low relative expression. **b** Venn diagrams indicate the number of total and overlapping lncRNAs with the significant differential expression in the *T. gondii* group, compared with the uninfected and inactivated groups, respectively. **c** and **d** Real time quantitative PCR (RT-qPCR) validation of the upregulated and downregulated lncRNAs (fold change ≥ 5, *P* < 0.05) from the microarray data

To explore the lncRNA expression profiles of HFF cells in response to *T. gondii* infection, microarray analyses were performed among the uninfected, inactivated *T. gondii-*treated and *T. gondii*-infected HFF cells. The inactivated group was used as a control to rule out the possibility that the host lncRNAs were regulated by the dead parasites. The lncRNAs with an expression change of more than two-fold were selected as differentially expressed candidates. We detected 6044 and 6436 lncRNAs in the *T. gondii*-infected group when compared with the uninfected group and the inactivated group individually (Table 1). Notably, the expression of 1206 lncRNAs (973 increased and 233 decreased) in the *T. gondii*-infected group displayed over five-fold changes, when compared with the uninfected group (Fig. 1b and Table 1, the list of lncRNAs is shown in Additional file 2: Table S2). There were 1316 differentially expressed lncRNAs (1022 increased and 294 decreased) with more than five-fold changes in the infected group when compared with the inactivated group (Fig. 1b and Table 1, the list of lncRNAs is shown in Additional file 3: Table S3). By only considering the lncRNAs that were actively regulated by *T. gondii* infection, 996 lncRNAs (800 upregulated and 196 downregulated) were selected as the significantly differentially expressed candidates (fold change ≥ 5) for the further study (Fig. 1a, b).

**Table 1:** Numbers of differentially expressed lncRNAs in the *T. gondii*-infected HFF cells

lncRNAs	*T. gondii* *vs* uninfected		*T. gondii* *vs* inactivated	
	FD ≥ 2.0	FD ≥ 5.0	FD ≥ 2.0	FD ≥ 5.0
Upregulated	4525	973	4873	1022
Downregulated	1519	233	1563	294
Total	6044	1206	6436	1316

*Abbreviations*: *T. gondii*, *T. gondii*-infected HFF cells; uninfected, uninfected HFF cells; inactivated, inactivated *T. gondii*-infected HFF cells; FD, fold changes

To validate the microarray data above, twenty lncRNAs (10 upregulated and 10 downregulated) were randomly selected from the candidates with significant differential expression for RT-qPCR detection. The RT-qPCR results showed that, after *T. gondii* infection, 9 of 10 upregulated lncRNAs and all of the 10 downregulated lncRNAs exhibited the same expression patterns as the microarray data (Fig. 1c, d).

### Differential expression of mRNAs after *T. gondii* infection

**Fig. 2:**
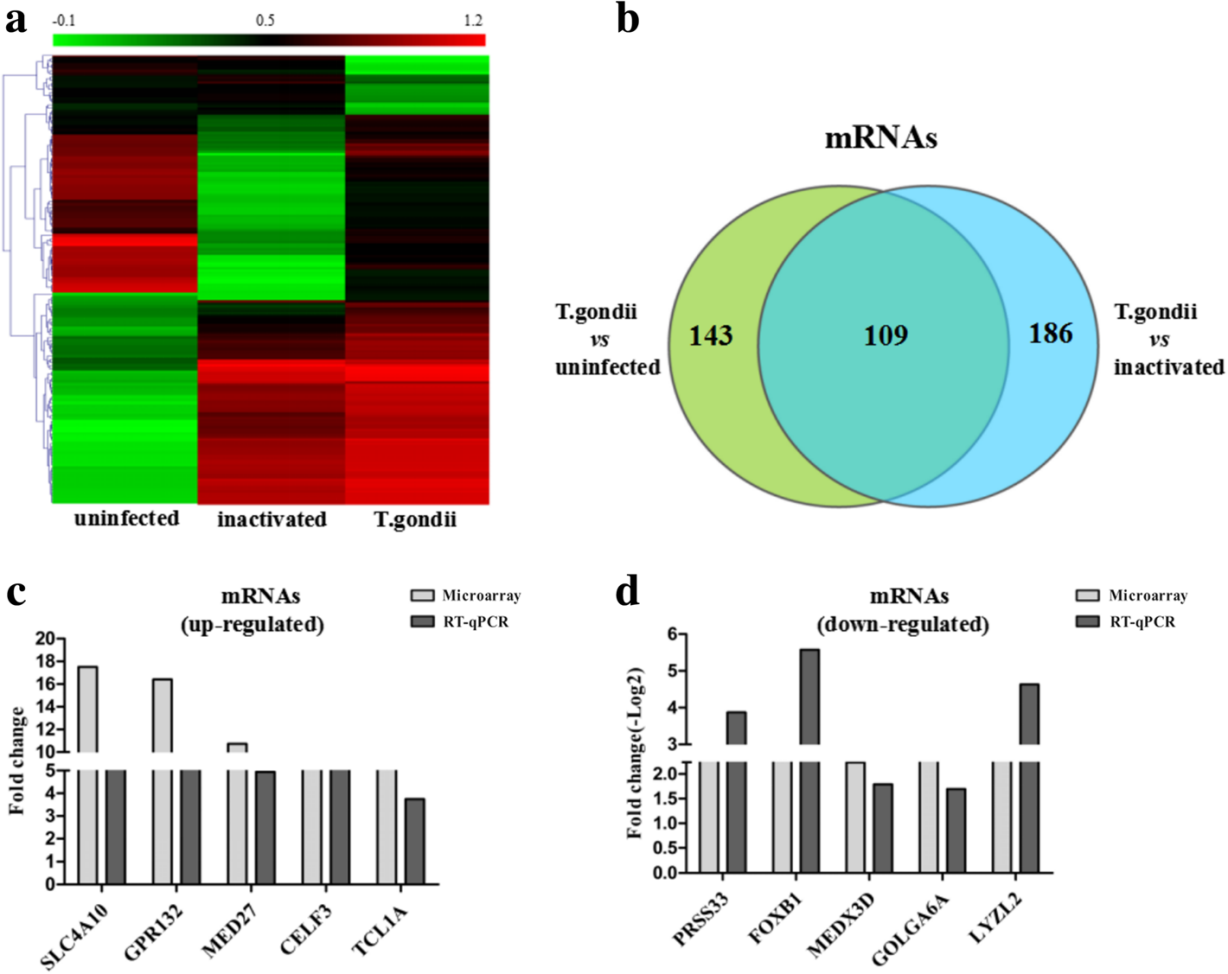
Differential expression of mRNAs after *T. gondii* infection. **a** The hierarchical clustering of significantly differentially expressed mRNAs (fold change ≥5, *P* < 0.05) between the uninfected HFF cells (uninfected), the inactivated *T. gondii*-infected HFF cells (inactivated) and the *T. gondii*-infected HFF cells (T. gondii). In the heat map, red indicates high relative expression, and green indicates low relative expression. **b** Venn diagrams indicate the number of total and overlapping mRNAs with significant differential expression in the *T. gondii* group compared with the uninfected and inactivated groups, respectively. **c** and **d** Real-time quantitative PCR (RT-qPCR) validation of the upregulated and downregulated mRNAs (fold change ≥5, *P* < 0.05) from the microarray data

Microarray analysis was also performed to explore the expression profiles of mRNAs in HFF cells after *T. gondii* infection. There were 1808 and 1924 mRNAs that showed differential expression (fold change ≥ 2) after *T. gondii* infection when compared with the uninfected group and the inactivated group, respectively (Table 2). Furthermore, we detected 143 and 186 mRNAs with a higher fold change (fold change ≥ 5) in the *T. gondii*-infected group compared with the uninfected and inactivated group, respectively (Table 2, the list of mRNAs is shown in Additional file 4: Table S4 and Additional file 5: Table S5). By excluding the host mRNAs that were regulated by the dead parasites, 109 mRNAs (82 upregulated and 27 downregulated) were selected as significant differential mRNA candidates for subsequent studies (Fig. 2a, b).

**Table 2:** Numbers of differentially expressed mRNAs in the *T. gondii*-infected HFF cells

mRNAs	*T. gondii* *vs* uninfected		*T. gondii* *vs* inactivated	
	FD ≥ 2.0	FD ≥ 5.0	FD ≥ 2.0	FD ≥ 5.0
Upregulated	1322	125	1427	159
Downregulated	486	18	497	27
Total	1808	143	1924	186

*Abbreviations*: *T. gondii*, *T. gondii*-infected HFF cells; uninfected, uninfected HFF cells; inactivated, inactivated *T. gondii*-infected HFF cells; FD, fold changes

To validate the mRNA expression from the microarray analyses, 10 mRNAs (5 upregulated and 5 downregulated) were randomly selected for RT-qPCR detection. The qPCR results showed that, in the *T. gondii-*infected HFF cell, 4 of 5 upregulated mRNAs displayed the same expression trends as the microarray data (Fig. 2c). For the downregulated mRNAs, 3 of 5 mRNAs exhibited more than a five-fold reduction after *T. gondii* infection (Fig. 2d).

### Gene ontology, KEGG pathway and co-expression network analyses

**Fig. 3:**
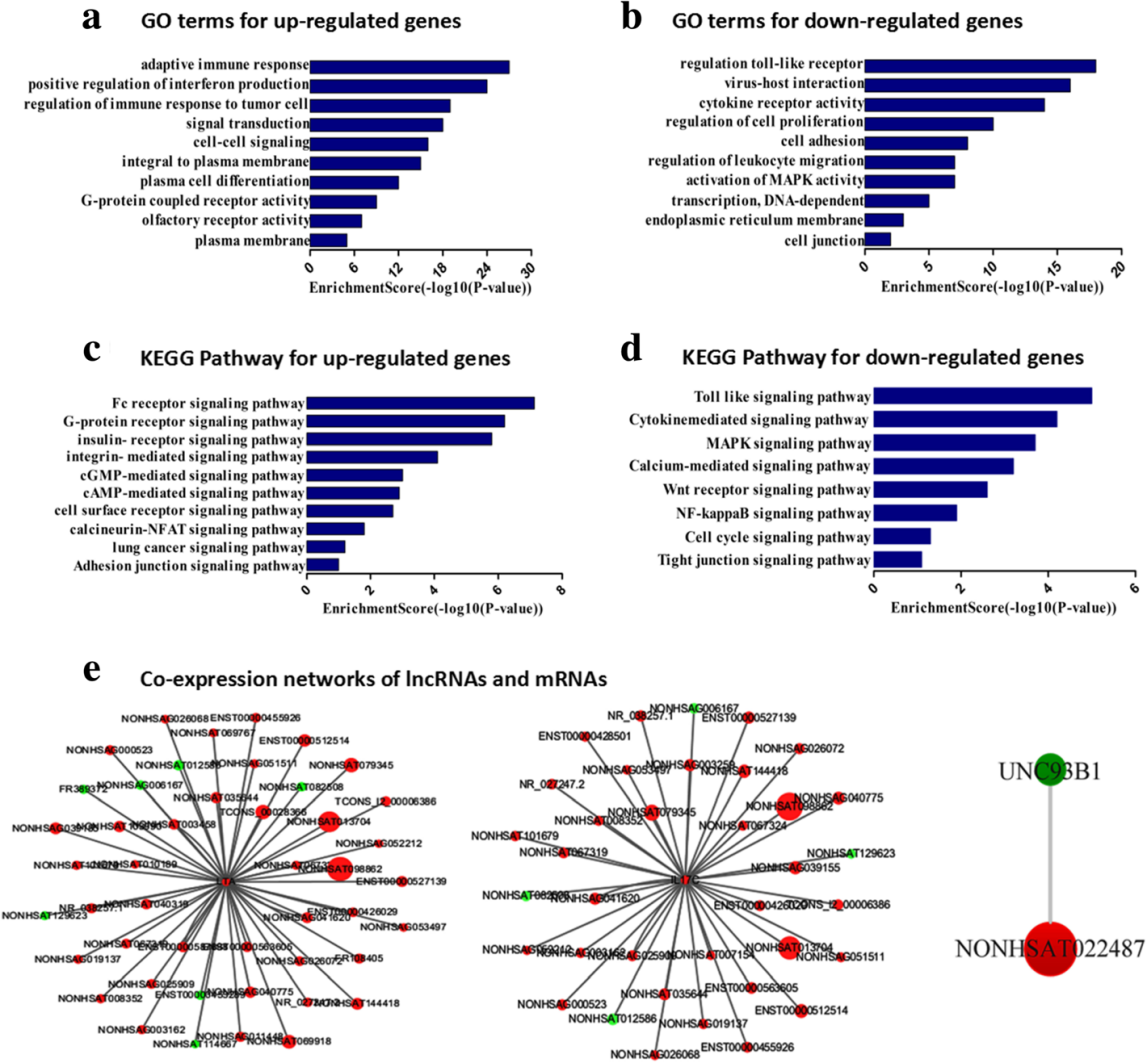
GO terms, KEGG pathway and Co-expression network analyses of differentially expressed mRNAs induced by *T. gondii* infection. The significant GO terms for the upregulated genes (**a**) and for the downregulated genes (**b**) in the HFF cells after *T. gondii* infection. The GO terms were filtered in accordance with *P* < 0.05 and FDR < 0.05. The most significant KEGG pathways for the upregulated genes (**c**) and for the downregulated genes (**d**) in the HFF cells after *T. gondii* infection. **e** The co-expression networks of the lncRNAs and mRNAs were constructed using Cytoscape software. The upregulated lncRNAs and mRNAs are shown by the red rectangle. The green rectangles represent the downregulated lncRNAs and mRNAs

Gene ontology (GO) analysis was carried out to predict the potential biological functions of the host mRNAs that were regulated by *T. gondii* infection. Based on the biological processes in the gene ontology classification, the significant differential mRNAs (fold change ≥ 5) from the microarray analyses were classified into different functional categories. The GO analysis showed that the three most enriched GO terms with upregulated mRNAs were the “adaptive immune response”, “positive regulation of interferon production” and “regulation of immune response to tumor cell” (Fig. 3a). The downregulated mRNAs were mainly involved in the “regulation toll-like receptor”, “virus-host interaction” and “cytokine receptor activity” (Fig. 3b). These results suggested that both upregulated and downregulated mRNAs were specifically correlated with the host immune response.

To further understand the predicted biological functions of the mRNAs with differential expression, the Kyoto Encyclopedia of Genes and Genomes (KEGG) pathway analysis was performed. The results indicated that the upregulated mRNAs mainly participated in the following signaling pathways: “Fc receptor signaling pathway”, “G-protein receptor signaling pathway” and “insulin-like receptor signaling pathway” (Fig. 3c). The downregulated mRNAs were significantly enriched in several immune signaling pathways, such as “Toll such as signaling pathway”, “Cytokine-mediated signaling pathway” and “MAPK signaling pathway” (Fig. 3d). These data indicated that the innate immune signaling in the host may be the main target regulated by *T. gondii* infection.

To determine the potential interaction between lncRNAs and immune-related genes during *T. gondii* infection, the coding-non-coding gene (CNC) co-expression network was constructed based on the correlation analysis. The lncRNAs were found to be involved in the expression of several genes coding for immune-related proteins, including LTA, IL17C and UNC93B1 (Fig. 3e). The additional co-expression network of lncRNAs and immune-related genes, such as IL-17RA, LST1, TNFRSF14 and AIF1, are shown in Additional file 6: Figure S1. For example, 39 lncRNAs, including NONHSAT144418, were positively correlated with the expression of the LTA gene, which encodes a protein of the tumor necrosis factor family; and 4 lncRNAs, including NONHSAG006167, were negatively correlated with the *Il* 17*c* gene coding for the cytokine IL17C that is restricted to activated T cells. Interestingly, we found that UNC93B1, which has been identified as one of the critical immune-related molecules in mediating the host defenses against *T. gondii* infection [35, 36], was correlated with a novel lncRNA, NONHSAT022487 (Fig. 3e). Additionally, the co-expression network analysis suggested that lncRNA NONHSAG006167 may be involved in the regulation of UNC93B1-related immune signaling.

### NONHSAT022487 suppress the expression of UNC93B1

**Fig. 4:**
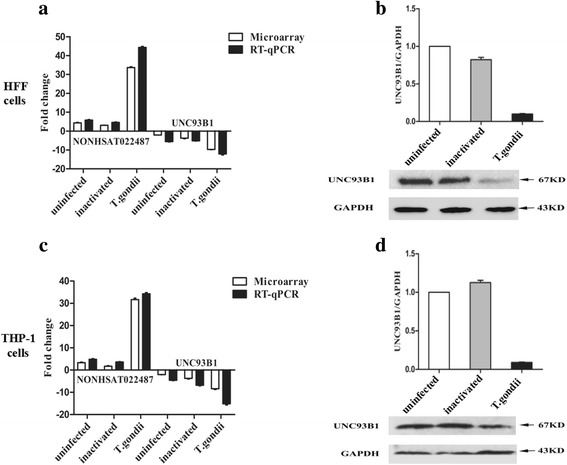
The expression patterns of NONHSAT022487 and UNC93B1 after *T. gondii* infection. **a** Microarray and RT-qPCR analyses showing the transcriptional levels of lncRNA NONHSAT022487 and the UNC93B1 gene in various HFF cells as indicated: uninfected, uninfected HFF cells; inactivated, inactivated *T. gondii*-infected HFF cells; *T. gondii*, *T. gondii*-infected HFF cells. **b** Western blot image showing the protein level of UNC93B1 in various HFF cells after *T. gondii* infection. GAPDH was used for internal normalization. The ratio of UNC93B1/GAPDH indicated the relative expression changes in various treated HFF cells according to Western blot. **c** As described in **a**, except that THP-1 cells were used. **d** As described in **b**, except that THP-1 cells were used

To examine the expression patterns of the lncRNA NONHSAT022487 and the mRNA UNC93B1, HFF and THP-1 cells were infected with *T. gondii*, respectively. The transcriptional levels of both NONHSAT022487 and UNC93B1 were analyzed by RT-qPCR. The protein expression of UNC93B1 was detected by Western blotting. Compared with both the uninfected and inactivated *T. gondii-*infected HFF cells, the transcriptional level of NONHSAT022487 was increased by more than 30-fold after *T. gondii* infection (Fig. 4a), whereas both mRNA and protein levels of UNC93B1 were decreased by over 10-fold in the *T. gondii-*infected HFF cells (Fig. 4a, b). The similar increased level of NONHSAT022487 transcription and decreased level of UNC93B1 expression were also observed in the *T. gondii*-infected THP-1 cells (Fig. 4c, d).

**Fig. 5:**
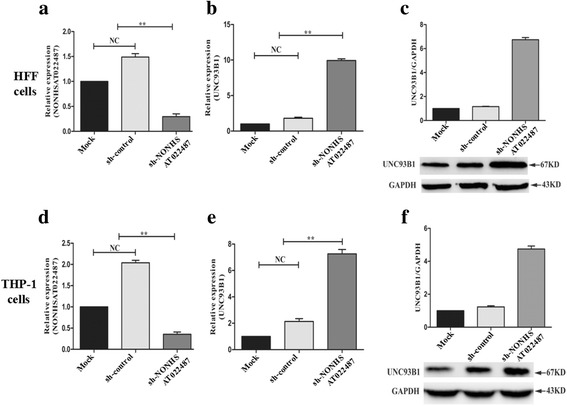
Upregulated expression of the UNC93B1 gene by knock down of NONHSAT022487. **a** RT-qPCR analysis showing the transcriptional levels of NONHSAT022487 in various HFF cells as indicated. Cells were transfected with the shRNA-NONHSAT022487 lentiviral plasmid (sh-NONHSAT02248) or shRNA-control plasmid (sh-control). **b** As described in **a**, except that the transcriptional level of the UNC93B1 gene was detected. **c** Western blotting image showing the protein expression of UNC93B1 in various HFF cells as indicated. GAPDH was used for internal normalization. The ratio of UNC93B1/GAPDH indicated the relative expression changes in various treated HFF cells according to Western blotting. **d** As described in **a**, except that THP-1 cells were used. **e** As described in **b**, except that THP-1 cells were used. **f** As described in **c**, except that THP-1 cells were used. Data are the mean with standard deviations (SD) from three independent biological replicates. *P < 0.05, **P < 0.01. *Abbreviations*: NC, no significant changes

To determine the regulation of lncRNA NONHSAT022487 on the expression of UNC93B1, NONHSAT022487 was knocked down in both HFF and THP-1 cells by transfection with the shRNA-NONHSAT022487 lentiviral plasmid (Additional file 7: Figure S2). Compared with the control plasmid, the relative transcriptional level of NONHSAT022487 was markedly reduced by 78.26% in HFF (*t*_(4)_ = 6.453, *P* = 0.0097) and 67.43% in THP-1 cells (*t*_(4)_ = 5.527, *P* = 0.0054) at 48 h post-transfection (Fig. 5a, d). In contrast, the relative transcriptional level of UNC93B1 was significantly increased by 5.5-fold in HFF (*t*_(4)_ = 5.013, *P* = 0.0068) and 3.75-fold in THP-1 cells (*t*_(4)_ = 4.761, *P* = 0.009) after knock-down of lncRNA NONHSAT022487 (Fig. 5b, e). The protein level of UNB93B1 was also increased by 6-fold in HFF (Fig. 5c) and 4-fold in THP-1 cells (Fig. 5f). These results indicated that knockdown of NONHSAT022487 significantly promoted the expression of the UNC93B1 gene.

**Fig. 6:**
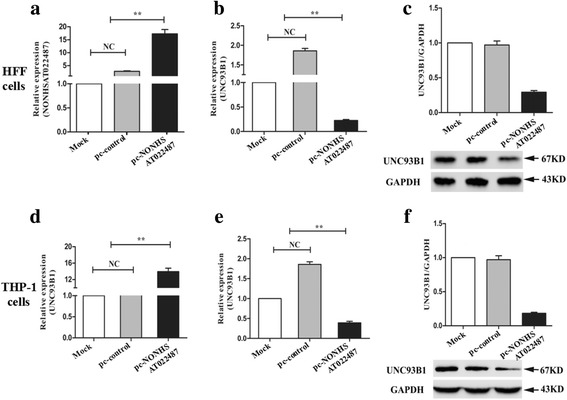
Downregulated expression of the UNC93B1 gene by over-expression of NONHSAT022487. **a** RT-qPCR analysis showing the transcriptional level of NONHSAT022487 in various HFF cells as indicated. Cells were transfected with pcDNA3.1-NONHSAT022487 (pc-NONHSAT022487) or pcDNA control plasmid (pc-control). **b** As described in **a**, except that the transcriptional level of UNC93B1 was detected. **c** Western blotting image showing the protein expression of UNC93B1 in various HFF cells as indicated, the GAPDH was used for the internal normalization. The ratio of UNC93B1/GAPDH indicated the relative expression changes in various treated HFF cells according to the Western blot. **d** As described in **a**, except that THP-1 cells were used. **e** As described in **b**, except that THP-1 cells were used. **f** As described in **c**, except that THP-1 cells were used. Data are the mean with standard deviations (SD) from three independent biological replicates. *P < 0.05, **P < 0.01. *Abbreviations*: NC, no significant changes

In addition, we over-expressed NONHSAT022487 in both HFF and THP-1 cells by the transfection of the pcDNA3.1 expression construct containing NONHSAT022487 into these cells. The relative transcription level of NONHSAT022487 was enhanced by 8-fold in HFF (*t*_(4)_ = 4.851, *P* = 0.008) and 10-fold in THP-1 cells (*t*_(4)_ = 5.218, *P* = 0.006) at 48 h post-transfection (Fig. 6a, d). As expected, the levels of transcription and protein expression of UNC93B1 were decreased by over 5-fold in both HFF ((*t*_(4)_ = 6.319, *P* = 0.0037) and THP-1 cells (*t*_(4)_ = 5.913, *P* = 0.0043) after over-expression of lncRNA NONHSAT022487 (Fig. 6b,c e, f), when compared with the control plasmid. These results indicated that the over-expression of lncRNA NONHSAT 022487 significantly inhibited the expression of UNC93B1 gene.

### NONHSAT022487 mediates the secretion of cytokines by negatively regulating UNC93B1 expression

**Fig. 7:**
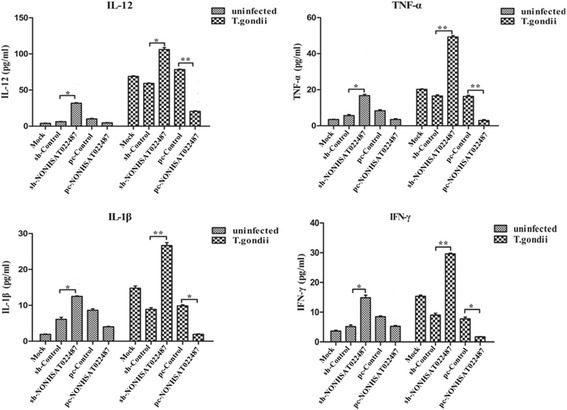
NONHSAT022487 mediates the secretion of cytokines in *T. gondii-*infected macrophages cells. The secretion of the cytokines IL-12, TNF-α, IL-1β and IFN-γ in the supernatant of various THP-1-derived macrophage cells from before (uninfected) or after *T. gondii* infection (T. gondii) were assessed by ELISA. Cells were transfected with shRNA-NONHSAT022487 lentiviral plasmid (sh-NONHSAT022487), shRNA control plasmid (sh-Control), pcDNA3.1-NONHSAT022487 plasmid (pc-NONHSAT022487) or pcDNA3.1 control plasmid (pc-Control) as indicated. Data are the mean with standard deviations (SD) from three independent biological replicates. *P < 0.05, **P < 0.01

UNC93B1 has been demonstrated to be critical for inducing cytokine production and autonomous control of *Toxoplasma* replication in macrophages [35, 36]. Since NONHSAT022487 can suppress the UNC93B1 expression, we wanted to examine the role of NONHSAT022487 in cytokine secretion. In the uninfected cells, the secretion levels of IL-12 (*t*_(4)_ = 3.305, *P* = 0.033), TNF-α (*t*_(4)_ = 3.417, *P* = 0.028), IL-1β (*t*_(4)_ = 3. 429, *P* = 0.027) and IFN-γ (*t*_(4)_ = 3.607, *P* = 0.023) were significantly upregulated after 48 h of transfection with shRNA-NONHSAT022487, when compared with the shRNA control (Fig. 7). Furthermore, these cells that over-expressed NONHSAT022487 exhibited a decrease in the secretion of cytokines, despite the lack of significance (Fig. 7).

After *T. gondii* infection, the production of cytokines IL-12, TNF-α, IL-1β and IFN-γ was enhanced in the infected cells, when compared with the uninfected cells. Especially for the *T. gondii* infected cells with transfection of shNONHSAT022487, the production of these cytokines was significantly enhanced when compared with those transfected with shRNA control (IL-12: *t*_(4)_ = 3.395, *P* = 0.03; TNF-α: (*t*_(4)_ = 7.923, *P* = 0.0014; IL-1β: *t*_(4)_ = 7.195, *P* = 0.0019; IFN-γ: *t*_(4)_ = 6.301, *P* = 0.0034). In contrast, over-expression of lncRNA NONHSAT022487 followed by infection with *T. gondii* impaired the secretion of the cytokines IL-1β (*t*_(4)_ = 2.721, *P* = 0.051) and IFN-γ (*t*_(4)_ = 3.015, *P* = 0.041), and a more significant inhibition of secretion was observed in IL-12 (*t*_(4)_ = 6.301, *P* = 0.0034) and TNF-α (*t*_(4)_ = 6.625, *P* = 0.0028) in these cells (Fig. 7). Collectively, these results suggested that the lncRNA NONHSAT022487 mediates the secretion of the cytokines IL-12, TNF-α, IL-1β and IFN-γ by negatively regulating the expression of UNC93B1 and that *T. gondii* infection can upregulate the expression of NONHSAT022487, thereby impairing the UNC93B1-regulated secretion of cytokines.

## Discussion

*Toxoplasma gondii* belongs to the phylum Apicomplexa, which contains many other important clinical and veterinary pathogens, such as *Plasmodium*, *Cryptosporidium* and *Eimeria* [37]. Since apicomplexans can only propagate within host cells, the regulatory cell biological function is critically important for their survival and pathogenesis [38]. Previous studies on the interaction of apicomplexan parasites and the host have largely focused on protein-coding genes [33, 39–41]. However, a number of recent studies have suggested that long ncRNAs (lncRNAs) were also involved in host-pathogens interactions [25, 27, 28, 42]. For instance, *Mycobacterium tuberculosis* (Mtb) infection altered the lncRNA and mRNA expression profiles in the human macrophage [42]. The analysis of the host transcription following SARS-CoV infection revealed that lncRNAs were associated with the host response to virus infection and altered innate immune signaling [27]. Our study of host long ncRNAs (lncRNAs) in response to *T. gondii* infection provides new insight into the regulating mechanism of the host gene expression by the Apicomplexan parasites.

In our study, the expression profiles of both lncRNAs and mRNAs in *T. gondii*-infected HFF cells were fully explored by microarray. Compared with the uninfected HFF cells, the expression profiles of both lncRNAs and mRNAs were profoundly altered in the *T. gondii*-infected HFF cells. The heat-killed parasite-infected HFF cells were used as a control to exclude the genes and pathways that were activated by dead parasites. There were redundant 392 lncRNAs and 116 mRNAs that were detected in the inactivated group compared with the uninfected group. They thus considered that the host lncRNAs and mRNAs were regulated by the dead parasites. A total of 996 lncRNAs and 109 mRNAs (fold change ≥ 5) were identified as the differential expression candidates and subsequently analyzed by GO term and KEGG pathway analysis. Interestingly, we found that the most enriched GO terms in the biological processes that targeted the upregulated genes are the adaptive immune response and the positive regulation of interferon production. However, the most enriched GO terms and KEGG pathways involved in the downregulated mRNAs are the Toll-like receptor and signaling pathways. It is believed that *T. gondii* possesses multiple means to down-modulate the production of pro-inflammatory cytokines [43]. One of the mechanisms for this downregulation is that *T. gondii* infection inhibits the intracellular signaling cascades, such as NF-κB and STAT1, within the infected cells [44, 45].

Toll-like receptors (TLRs) are pathogen-associated molecular pattern (PAMP) recognition receptors that play a central role in macrophage activation and the control of parasitic infections [36, 46]. The *T. gondii-*derived component profilin can be recognized by TLR11and TLR12 and induce IL-12 production that limits the cyst burden in *T. gondii*-infected mice [47, 48]. The UNC93B1, a multi-transmembrane endoplasmic reticulum-resident protein, is required for proper TLR localization and signaling in both humans and mice [49, 50]. UNC93B1 can facilitate trans-location of TLR (TLR3, TLR7 and TLR9) from the endoplasmic reticulum to the Golgi [51, 52] and mediates the host immune responses to infection with murine cytomegalovirus [53], as well as several intracellular protozoan parasites [35, 54]. A deficiency in the functional UNC93B1 protein abolished TLR dependent cytokine secretion by macrophages and attenuated immune responses against intracellular parasite infection, including *Trypanosoma cruzi* [54] and *T. gondii* [35, 36]. Mice lacking functional UNC93B1 result in the impaired production of IL-12, IFN-γ and enhanced *T. gondii* tachyzoite replication [35, 36]. However, the role of UNC93B1 in intracellular parasite growth appears to be independent of TLR function [35].

Our results suggested that NONSHAT022487 can suppress the expression of the immune-related molecule UNC93B1 by lncRNA knockdown and over-expression experiments. Meanwhile, the RT-qPCR results in Figs. 5 and 6 indicate that there is a non-significant difference in the expression of NONHSAT022487 and UNC93B1 between the mock and the controls. Considering that both lncRNA NONHSAT022487 and UNC93B1 are involved in innate immune responses, it is possible that their different expression maybe affected by nonspecific treatment, such as transfection with GFP shRNA and pcDNA empty vector. Our further study also demonstrated that NONSHAT022487 can regulate the secretion of the cytokines IL-12, TNF-α, IL-1β and IFN-γ after *T. gondii* infection. To survive successfully, the *Toxoplasma* parasite actively regulates the host immune response to downregulate the production of proinflammatory cytokines, which could be harmful to the parasite [15, 32]. For example, *T. gondii* infection inhibits the host cell transcriptional response to IFN-γ [55]. Meanwhile, *T. gondii-*infected murine splenic lymphocytes could impair the capacity of these cells to produce cytokines and immunoglobulin secretions [43]. This regulation of the host immune response is critical for the parasite to establish a chronic infection. On the other hand, the host has to downregulate the immune responses shortly after initial upregulation to control damages to self. Several lines of emerging evidence have indicated that lncRNAs function in the regulation of the mammalian innate and adaptive immune responses [23, 56]. For example, lncRNA-Cox2 has been identified to regulate the TLR signaling pathway [56], while lncRNA PACER (p50-assocated COX-2 extragenic RNA) acts as a decoy molecule in the NF-κB signaling pathway [23]. Therefore, further studies will be needed to elucidate the role of lncRNAs in *Toxoplasma* infection and immune regulation.

## Conclusions

In this study, we investigated the differential expression profiles of lncRNAs and mRNAs in *T. gondii* infected-HFF cells and revealed that the differential expressed genes were mainly involved in the host immune response. Significantly, we identified the lncRNAs as regulatory molecules involved in the host immune signaling to *T. gondii* infection, which advances our understanding of the interaction of *T. gondii* parasites and host cells.

## Additional files

Additional file 1: Table S1. A list of primer sequences used for the real-time qPCR detection. (XLSX 11 kb)

Additional file 2: Table S2. A list of significantly differentially expressed lncRNAs between the *T. gondii* infected and uninfected groups. (XLSX 63 kb)

Additional file 3: Table S3. A list of significantly differentially expressed lncRNAs between the *T. gondii* infected and inactivated groups. (XLSX 68 kb)

Additional file 4: Table S4. A list of significantly differentially expressed mRNAs between the *T. gondii* infected and uninfected groups. (XLSX 15 kb)

Additional file 5: Table S5. A list of significantly differentially expressed mRNAs between the *T. gondii* infected and uninfected groups. (XLSX 17 kb)

Additional file 6: Figure S1. The additional interaction network of lncRNAs and immune-related genes induced by *T. gondii* infection. (TIFF 1644 kb)

Additional file 7: Figure S2. Transfection of shRNA-NONHSAT022487 into HFF and THP-1 cells. (TIFF 972 kb)

## Abbreviations

HFF cells: human foreskin fibroblast cells
inactivated: inactivated *T. gondii-*treated cells
infected: *T. gondii-*infected cells
lncRNAs: long non-coding RNAs
MOI: multiplicity of infection
PV: parasitophorous vacuole
RT-qPCR: real-time quantitative PCR
uninfected: uninfected cells
