# Supplementary figures and images for: Microarray analysis of long non-coding RNA expression profiles uncovers a Toxoplasma-induced negative regulation of host immune signaling
Source: Parasit Vectors. 2018 Mar 12;11:174. doi: 10.1186/s13071-018-2697-8 (PMC5848448; doi:10.1186/s13071-018-2697-8)

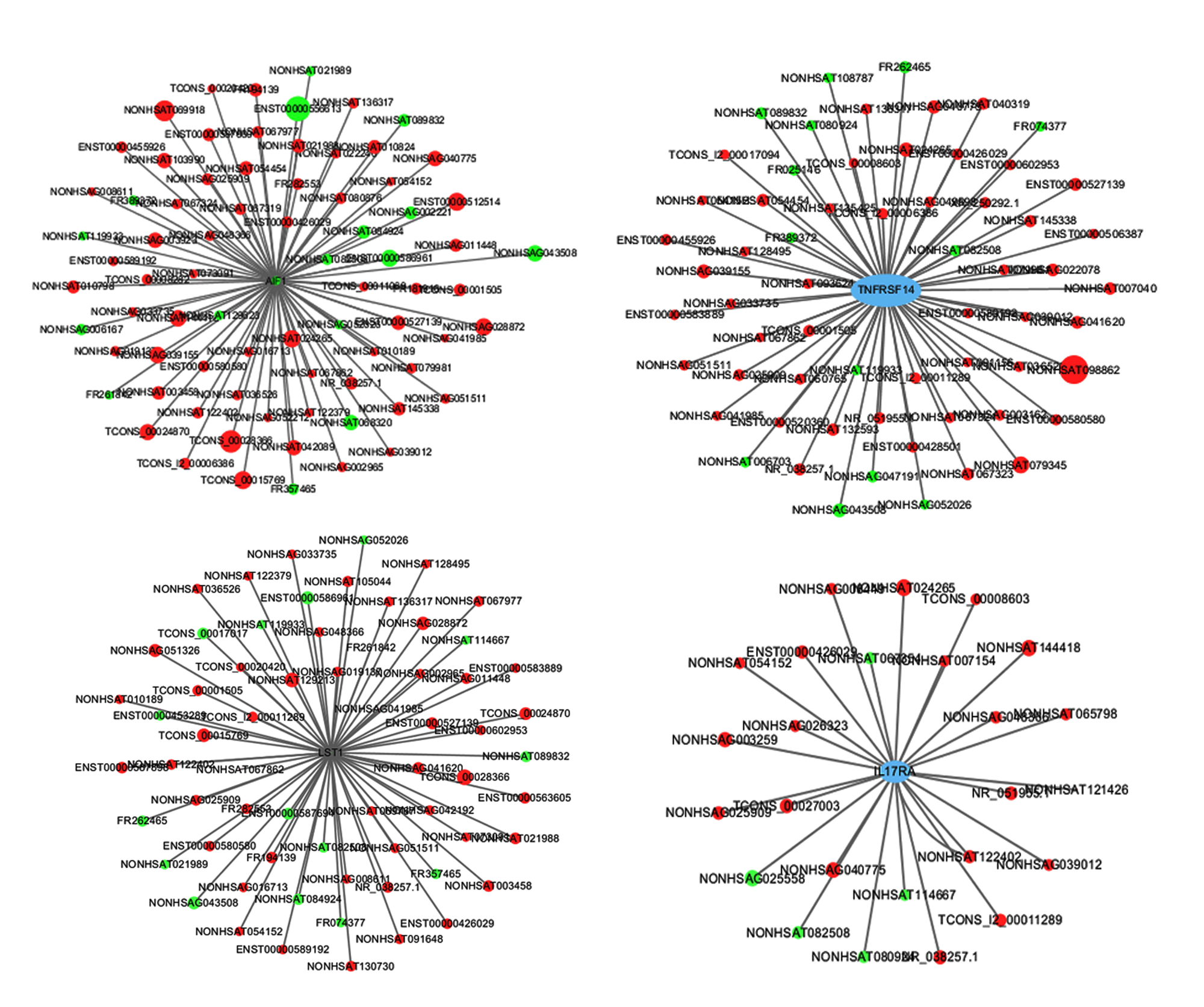

Supplement: Supplementary file 6 — The additional interaction network of lncRNAs and immune-related genes induced by T. gondii infection. (TIFF 1644 kb) [file 13071_2018_2697_MOESM6_ESM.tif]

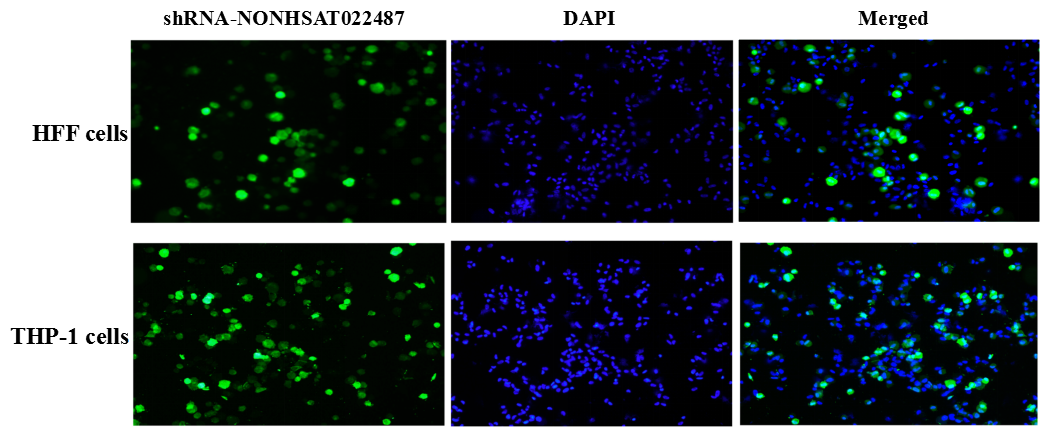

Supplement: Supplementary file 7 — Transfection of shRNA-NONHSAT022487 into HFF and THP-1 cells. (TIFF 972 kb) [file 13071_2018_2697_MOESM7_ESM.tif]
